# Click chemistry, 3D-printing, and omics: the future of drug development

**DOI:** 10.18632/oncotarget.6787

**Published:** 2015-12-29

**Authors:** Razelle Kurzrock, David J. Stewart

**Affiliations:** ^1^ Center for Personalized Cancer Therapy and Division of Hematology and Oncology, University of California San Diego Moores Cancer Center, San Diego, CA, USA; ^2^ Division of Medical Oncology, University of Ottawa, Ottawa, ON, Canada

**Keywords:** omics, drug development, anti-cancer drugs, genomics, 3D

## Abstract

Genomics is a disruptive technology, having revealed that cancers are tremendously complex and differ from patient to patient. Therefore, conventional treatment approaches fit poorly with genomic reality. Furthermore, it is likely that this type of complexity will also be observed in other illnesses. Precision medicine has been posited as a way to better target disease-related aberrations, but developing drugs and tailoring therapy to each patient's complicated problem is a major challenge. One solution would be to match patients to existing compounds based on *in silico* modeling. However, optimization of complex therapy will eventually require designing compounds for patients using computer modeling and just-in-time production, perhaps achievable in the future by three-dimensional (3D) printing. Indeed, 3D printing is potentially transformative by virtue of its ability to rapidly generate almost limitless numbers of objects that previously required manufacturing facilities. Companies are already endeavoring to develop affordable 3D printers for home use. An attractive, but as yet scantily explored, application is to place chemical design and production under digital control. This could be accomplished by utilizing a 3D printer to initiate chemical reactions, and print the reagents and/or the final compounds directly. Of interest, the Food and Drug Administration (FDA) has recently approved a 3D printed drug—levetiracetam—indicated for seizures. Further, it is now increasingly clear that biologic materials—tissues, and eventually organs—can also be “printed.” In the near future, it is plausible that high-throughput computing may be deployed to design customized drugs, which will reshape medicine.

## INTRODUCTION

Thousands of people are taking medications that will not help them or may harm them. Indeed, the top ten best-selling drugs in the United States are only effective in between 4% and 25% of the individuals for whom they are prescribed [1]. In contrast, precision medicine implies fitting therapy to the distinct molecular biologic features of each patient and their illness. Precision medicine is most advanced in the cancer field, but will undoubtedly be applicable across medical specialties.

In oncology, the molecular biologic features that define a cancer may include a wide range of genomic, transcriptomic, and/or proteomic variables that drive the tumor or form the signature of the host environment, including, but not limited to, the immune response. The deployment of precision oncology is being enabled by breathtaking technological progress in genomic sequencing, as well as the increasing availability of targeted and immunotherapeutic compounds. Yet, next generation sequencing may be a disruptive technology in that its results suggest that canonical models of clinical research and practice are a poor fit with the complex reality unveiled in metastatic cancers. Indeed, it is apparent that tumors and their hosts have remarkably heterogeneous molecular landscapes that differ from individual to individual [2–4].

Traditional models of clinical research and practice are drug centered, with the approach of ascertaining commonalities between patients so that they can be grouped together and treated in the same way. However, if each patient has a unique omic landscape, a new patient-centered, N-of-one strategy that prosecutes cancer with individually tailored treatments is needed [1–4]. This complexity is likely to also apply beyond cancer to other aspects of medicine. Therapy optimization will conceivably necessitate designing compounds for individual patients with the use of *in silico* modeling, and technology for just-in-time production, perhaps realizable soon by three-dimensional (3-D) printing.

Fortunately, technological advances are occurring at a startling pace in 3D printing, material design, and bio-printing, as well as omics-based diagnostics, computer modeling of 3D crystal structure for molecular aberrations and chemicals, and 3D drug printing. Indeed, there is now a large body of work in the literature that deals with very different, specific 3D printing techniques. While the specific advantages and disadvantages of different materials and techniques are a matter of debate, the quick synthesis of new chemicals or drugs based on computer simulations is likely to become feasible in the near future, and may enable customization of complex therapies to individual patients.

3D-Printing Defined: 3D-printing is the process of making a three-dimensional object of practically any shape from a digital model. 3D-printing is generally realized using an additive manufacturing process, which means that successive layers of material are laid down in different shapes. It works by building a solid object from a series of layers—each one printed directly on top of the previous one. It diverges from traditional ways of building objects, which often rely on subtractive processes—that is cutting or drilling in order to remove or sculpt material.

The process of 3-D manufacturing takes virtual blueprints from computer-based drafts and segments them into digital cross-sections for the machine to successively use as a guideline for printing. Sequential layers of assorted materials are deposited in order to construct the object from a series of cross sections. These cross sections correspond to the virtual slices of the model in the computer files. After the layers are placed, they are fused. When the process is finished, a final 3D model has been “printed.” Theoretically, in a few hours, perhaps less in the future, this technique can create almost any object.

3D-Material Design Printing: Harnessing supercomputers and the equations of quantum mechanics, it is possible to design new materials atom by atom, without ever performing an experiment [5, 6]. The technique is termed high-throughput computational materials design. The concept is straightforward—-use supercomputers to virtually analyze thousands of chemical compounds, quickly and efficiently seeking out the best building blocks for novel materials.

Most materials are made of many chemical compounds. However, regardless of a material's complexity, its properties are determined by the characteristics of its atoms. The first step in high-throughput materials design is to virtually create new materials by making thousands of quantum-mechanical calculations. A supercomputer positions virtual atoms into thousands of simulated crystal structures. It then determines the properties of the virtual compounds, and screens them based on those characteristics deemed desirable.

Researchers that aim to hasten the computer-driven materials revolution already exist and collaborate. Objectives include building open-access databases that elaborate the fundamental thermodynamic and electronic characteristics of all known inorganic compounds [5]. To date, the basic properties of nearly all of the approximately 35,000 inorganic materials known to exist in nature, as well as a few thousand materials that are theoretical, have been delineated [5].

3D-bioprinting: 3D printing is beginning to be exploited in tissue engineering applications in which organs and body parts are produced [7, 8, 9]. Several designations have been employed to signify this field of research: bio-printing, organ printing, body-part printing, and computer-aided tissue engineering. In the process of 3D-bioprinting, layers of living cells are deposited onto an appropriate matrix, and gradually assembled to form three-dimensional structures including vascular systems.

There are many applications for 3D bio-printing. As an example, it has been established that it is possible to produce customized food with 3D Hydrocolloid Printing [10, 11], though many challenges in regard to materials and construction remain. It has also been recently demonstrated that 3D printing can produce bone grafts with localized organic bioactive loading and diffusion control, possibly offering a breakthrough for patients by delivering the necessary material function to match human bone health status, site of repair, and age [12].

Click Chemistry and 3D drug printing: Click chemistry was a term coined to address the possibility of developing synthetic strategies that enable much more rapid discovery and production of molecules with a desired profile of properties [13]. Herein, we have adapted the term to infer the quick synthesis of new chemical compounds or drugs based on computer simulations. Nature's molecules are fashioned from a small set of building blocks using a few types of reactions. Supercomputers can model natural proteins, including their 3D crystal structures, and create drugs/chemicals *in silico* that interact with them. Once the 3D structure of a protein is fully visualized, and the effect of the drug modeled, 3D printers and click chemistry procedures would allow generation of the appropriate compound. Indeed, proof-of principle, prototype projects demonstrated that it is possible to use 3D-printing techniques to generate and manufacture chemical compounds [14, 15, 16].

In applying this technology to drugs, the first generation technology is already a reality. Indeed, recently, the US Food and Drug Administration (FDA) approved the 3D-printed oral drug product—*Spritam* (levetiracetam) (Aprecia Pharmaceuticals) [17].

Levetiracetam is indicated as adjunctive treatment for seizure disorders. It was developed with proprietary technology, which uses 3D printing to create a porous formulation of the antiepileptic that disintegrates rapidly with a sip of liquid, overcoming the difficulty patients with swallowing disorders or children have in taking large pills.

It has been suggested that the second-generation efforts will involve getting a digital prescription, buying the “blueprint” and chemical “materials” needed, and then printing the drug at home with the software and a 3D molecular printer [15]. Hence, 3D drug printing will have important repercussions in the realm of distribution of medicines. However, even more profound are the eventual implications for new drug discovery and personalized therapy. Indeed, third-generation 3D drug printing would entail the creation of new drugs that maximize efficacy and minimize toxicity. In cancer, optimization might be based on the known “omic” aberrations in a patient's tumor, as well as the host's genomic composition. Supercomputers could tailor chemicals and dosages to the specific needs of an individual. It would not be the drugs themselves that would need to be sold, but rather the software that permits their design and generation.

One can envision that, within a very few decades, next generation sequencing (or another innovative technology) will supplant biopsies and light microscopy as a method of characterizing nodules with respect to presence of malignancy, tumor type, potentially actionable abnormalities and impending resistance mechanisms. Similar diagnostic techniques might be developed for almost every branch of medicine. Computer algorithms will define the 3D shape of the targets, and the shape of the drug molecule that would most likely be required to inhibit or bind to the target while having optimal characteristics for oral absorption and tissue penetration, and while carrying minimal risk of normal tissue toxicity.

## SUMMARY AND FUTURE DIRECTIONS

Alchemy was a medieval chemical discipline that aimed to attain the transmutation of the base metals into gold, the identification of a universal cure for illness, and the discovery of the key to eternal life. More colloquially, it implied a process of transforming a common object, element, or material into something unique and desirable. Though alchemy was long ago discredited, supercomputers may soon provide the capability to create materials and medicines in a manner comparable to that envisioned by the alchemists.

3D printing may have significant impact on our lives, from the generation of commonplace appliances to the possibility of food replicators. Of interest, 3D printing is already being used to produce a variety of medical devices, with mass customization enabled by this technique. Examples include individually tailored dental prosthetics, prescription eyeglasses, and hearing aids.

Chemicals can also be 3D printed. Indeed, the FDA has already approved a 3D printed drug [17]. It therefore appears inevitable that we may be able to soon routinely 3D print drugs, including customized medications [15]. In cancer, for instance, it is plausible that integrated omics profiling could reveal the specific driver aberrations in a tumor, as well as host vulnerability to toxicity. Computer modeling, including a deep interrogation of crystal structures, and use of quantum mechanics-based understanding of the fundamental behavior of matter, could then be exploited to calculate the drug design most likely to be effective while minimizing side effects. Computer simulations would be based on the process by which molecular compounds operate within the body. For instance, some targeted pharmaceuticals work because they bind specifically to certain cellular receptors that are implicated in disease [18]. Interestingly in this regard, the Oak Ridge Leadership Computing Facility recently simulated, in less than two days, screening of two million different drug compounds, by examining 3D biological structures of candidate agents docking with targeted receptors [19, 20, 21].

In the future, the “prescription” resulting from supercomputer modeling would be used to instruct the 3D printer to properly print the drug. The 3D drug printer and click chemistry methods that are rapidly evolving will then quickly synthesize the drug(s) required for the patient's personal use. Undoubtedly there are still numerous challenges to creating custom-designed drugs, and advances in this field would also be disruptive to current regulatory and commercial standards. Even so, as the technology for 3D printing of drugs develops, it is conceivable that there would be a shift to regulatory oversight of the quality of computer algorithms, which would also be the source of intellectual property. Further technological innovations are necessary before these visions can be realized, but it seems likely that the ability to interrogate and prosecute human disease will be transformed, in the foreseeable future, by advances in omics profiling, computer modeling, and 3D chemical printing.
